# A Unique SUMO-Interacting Motif of Trx2 Is Critical for Its Mitochondrial Presequence Processing and Anti-oxidant Activity

**DOI:** 10.3389/fphys.2019.01089

**Published:** 2019-08-27

**Authors:** Chaofei Chen, Kang Wang, Haifeng Zhang, Huanjiao Jenny Zhou, Yuxin Chen, Wang Min

**Affiliations:** ^1^Center for Translational Medicine, The First Affiliated Hospital, Sun Yat-sen University, Guangzhou, China; ^2^Department of Pathology, Vascular Biology and Therapeutics Program, Yale School of Medicine, Yale University, New Haven, CT, United States; ^3^Department of Laboratory Medicine, Nanjing Drum Tower Hospital, Nanjing University Medical School, Nanjing, China

**Keywords:** thioredoxin-2, mitochondria, sumoylation, SUMO-interacting motif, senescence

## Abstract

**Objective:**

Mitochondrial thioredoxin 2 (Trx2) is a vital mitochondrial redox protein that mediates normal protein thiol reduction and provides electrons to peroxiredoxin 3 (Prx3) to scavenge H_2_O_2_ in mitochondria. It has been widely reported that Trx2 deletion in cells or mice generates massive reactive oxygen species (ROS) which have been implicated in many pathological processes. On the contrary, how ROS regulate Trx2 processing and activity remains to be elucidated.

**Approach and Results:**

Here we show that excess ROS induce endothelial cell senescence concomitant with an attenuation of Trx2 processing in which Trx2 presequence [i.e., mitochondrial targeting signal peptide (MTS)] is cleaved to generate a mature form. Mutation analyses indicate that Trx2 processing is mediated by mitochondrial processing peptidase (MPP) and mitochondrial intermediate peptidase (MIP)-recognition sites within the MTS. Interestingly, a mutation at a SUMO- interacting motif (SIM), but not the catalytic sites within the mature Trx2 protein, completely blocks Trx2 processing with no effect on Trx2 mitochondrial targeting. Consistently, chemical inhibition of protein SUMOylation attenuates, while SUMOylation agonist promotes, Trx2 processing. Moreover, we identify the α–MPP subunit is a SUMOylated protein that potentially mediates Trx2-binding and cleavage. Furthermore, the unprocessed form of Trx2-SIM is unable to protect cells from both ROS generation and oxidative stress-induced cellular senescence.

**Conclusion:**

Our study reveals that a unique SUMO-interacting motif of Trx2 is critical for its mitochondrial processing and subsequent anti-oxidant/antisenescence activities.

## Highlights

- Senescence stimuli induce endothelial cell senescence concomitant with an attenuation of mitochondrial Trx2 processing, resulting in accumulation of Trx2 precursor (PreTrx2).
- Trx2 processing is mediated by MPP and MIP-recognition sites within the mitochondrial targeting sequence of Trx2.
- A unique SUMO-interacting motif of Trx2 is critical for its mitochondrial processing and subsequent anti-oxidant/anti-senescence activities.

## Introduction

Cardiovascular diseases (CVD) are and will remain the leading cause of global death and disability (Benjamin et al., 2017; Xue et al., 2017). CVD mortality rates increase with age, and the aging process poses the largest risk factor for the development of CVD (Finegold et al., 2013). Chronic exposure to CVD risk factors increases oxidative stress, which hastens the development of vascular endothelial senescence that could contribute to the pathogenesis of CVD (Voghel et al., 2007).

Vascular endothelial cells senescence has been increasingly linked to both aging and age-related diseases, including heart failure, diabetes, and atherosclerosis (Minamino and Komuro, 2007; Voghel et al., 2007; Tian and Li, 2014; Katsuumi et al., 2018). Stress-induced premature senescence (SIPS) is induced by oxidative stress, oncogene activity, or suboptimal culture conditions, which occurs independently of a change of telomere length (Coleman et al., 2010). The senescent cells exit the cell cycle but remain viable, display a flattened and enlarged morphology, and show an accumulation of senescence associated β-galactosidase (SA-β-Gal) activity (Dimri et al., 1995). Senescence is occurred via p53 pathway which transactivates the cyclin-dependent kinase inhibitor p21^WAF/CIP1^ or through the p16^INK4a^(p16) pathway (Coleman et al., 2010). In addition, DNA damage marker phosphorylation of γH2A.X can also be used to identify senescent cells (Munoz-Espin and Serrano, 2014).

Many senescence-associated vascular diseases are associated with the generation of reactive oxygen species (ROS) (Erusalimsky, 2009). Different types of stresses, including chemotherapeutic drugs, loss of telomeric protective functions, DNA damage and oncogene activation lead to the increase of ROS (Munoz-Espin and Serrano, 2014). Mechanistically, high intracellular ROS induces the activation of p16 or p53/p21 through MPAKK3 and MAPKK6, and their downstream kinase effector p38, eventually activating cell cycle inhibitors and the tumor suppressor RB (Munoz-Espin and Serrano, 2014). Of the functional ROS, the oxidative stressor hydrogen peroxide (H_2_O_2_) is often used to induce an oxidative environment that rapidly leads to premature senescence (Oeseburg et al., 2010; Huo et al., 2018).

Thioredoxin 2 (Trx2) is a key mitochondrial redox protein that balances the ROS levels and maintains mitochondrial function in various cells (Hirasaka et al., 2011; Li et al., 2014, 2017; Huang et al., 2015; Holzerova et al., 2016; Chen et al., 2017). The mitochondrial Trx system consists of thioredoxin reductase 2 (TrxR2), Trx2 and peroxiredoxin 3 (Prx3) (Spyrou et al., 1997; Nonn et al., 2003). This system using NADPH maintains normal protein function and also provides electrons to PRX3 to scavenge H_2_O_2_ in mitochondria. Trx2 is a critical regulator of redox balance, which protects cells from oxidative stress-induced apoptosis (Tanaka et al., 2002; Li et al., 2014). To date, the role of Trx2 in vascular endothelial cells senescence has not been investigated.

Many mitochondrial precursor proteins contain a N-terminal pre-sequence, i.e., mitochondrial targeting signal peptide (MTS). MTS is often proteolytically by the mitochondrial processing peptidase (MPP). Subsequently additional cleavages can occur to remove newly exposed N-terminal sorting peptides by mitochondrial intermediate peptidase (MIP) or inner membrane peptidase (IMP) to yield the mature proteins (Gakh et al., 2002; Carapito et al., 2017). Some mitochondrial proteins contain a sorting signal outside of the MTS (Monaghan and Whitmarsh, 2015). Trx2 also contains conserved motifs that might be recognized and cleaved by MPP, MIP and IMP (Hirasaka et al., 2011). Whether these motifs play an important role in Trx2 processing remains to be elucidated.

Sequence analyses indicate that Trx2 contains a unique SUMO-interacting motif (SIM), characterized as a short stretch of hydrophobic amino acids, consensus Val/Ile-X-Val/Ile-Val/Ile (V/I-X-V/I-V/I), flanked by acidic residues (Gareau and Lima, 2010). SIMs mediate non-covalent binding of SIM-containing proteins and proteins with SUMOylation (Kerscher, 2007), a reversible posttranslational modification. Similar as ubiquitination, SUMOylation targets at a specific lysine (K) via the small ubiquitin-related modifier (SUMO) protein family. SUMOylation is a dynamic process that is mediated by a heterodimeric SUMO activating enzyme E1, a signal conjugating enzymes E2, and ligating enzymes E3 and is readily reversed by a six-member family of SUMO-specific proteases (SENPs) (Yeh, 2009). SUMOylation is considered to be an essential process that modulates various critical cellular processes, including DNA replication, transcription, DNA damage repair, nuclear trafficking, cell cycle regulation, pre-mRNA splicing, signal transduction, protein-protein, and protein-DNA interaction (Pichler et al., 2017). Multiple lines of evidence have shown that SUMOylation and SIMs regulates cellular senescence (Sahin et al., 2014a, b). In the vasculature, SUMOylation is significantly involved in oxidative stress and EC function (Qiu et al., 2017; Zhu et al., 2017; Zhou et al., 2018). However, the role of SUMOylation in EC senescence has not been investigated.

In the present study, we unexpectedly reveal that unique SUMO-interacting motifs of Trx2 is critical for Trx2 mitochondrial processing and its subsequent anti-oxidant/ antisenescence activities.

## Materials and Methods

### Chemicals and Antibodies

Ginkgolic Acid [(GA,15:1) - CAS 22910-60-7, 345887] was purchased from Millipore. N-ethylmaleimide (NEM, SENPs inhibitor, E1271), streptonigrin (SN, SENPs inhibitor, S1014) were bought from Sigma. Protein A/G PLUS-Agarose (sc-2003) was obtained from Santa Cruz.

Antibodies for immunoblotting, immunoprecipitation, and immunostaining were: V5 (Cell Signaling Technology, 13202, Rabbit, WB 1:1000), Trx2 (Abcam, ab185544, Rabbit, WB, 1:10000), Trx2 (Santa Cruz, sc-133201, Mouse, IF, 1:50), Phospho-p53 (Cell Signaling Technology, 9284, Rabbit, WB 1:500), P21 (Cell Signaling Technology, 2947, Rabbit, WB 1:1000), Phospho-Histone H2A.X (Cell Signaling Technology, 9718, Rabbit, WB 1:200), β-Actin (Cell Signaling Technology, 4970, Rabbit, WB 1:1000), α/β-Tubulin (Cell Signaling Technology, 2148, Rabbit, WB 1:1000), TFAM (Cell Signaling Technology, 8076, Rabbit, IF 1:100), PMPCA (Santa Cruz, sc-390471, mouse, IP 1:50 WB 1:500), SUMO1 (Cell Signaling Technology, 4930, Rabbit, WB 1:200), SUMO2/3 (life technologies, 519100, Rabbit, 1:600), Donkey anti-Mouse IgG (H + L) Highly Cross-Adsorbed Secondary Antibody, Alexa Fluor 488 (Thermo Fisher Scientific, A-21202, IF 1:200), Donkey anti-Rabbit IgG (H + L) Highly Cross- Adsorbed Secondary Antibody, Alexa Fluor 594 (Thermo Fisher Scientific, A-21207, IF 1:200), normal mouse IgG (Santa Cruz, sc-2025, IP 1:100).

### Cell Culture, Transfection, siRNA Transfection, and Lentivirus Gene Expression

HEK293T and HeLa cells were cultured in DMEM containing 10% fetal bovine serum (FBS). Primary human umbilical vein endothelial cells (HUVECs) were purchased from the tissue culture core of the Yale Vascular Biology and Therapeutics (VBT) Program and maintained in EBM-2 (Lonza) supplemented with 2% FBS, 2 mM L-glutamine, penicillin/streptomycin, and EGM-2 growth factors. All the cells were maintained at 37°C incubator with 5% CO_2_ atmosphere.

Lipofectamine 3000 was used to transfect 293T or Hela cells according to the manufacture’s protocol (Life Technologies). Cell were cultured in 12-well plates until they reached 60–80% confluence at which time they were transfected with a total of 1 μg plasmids. Cells were treated and harvested for protein assays or immunofluorescence at 48 h after transfection.

Lipofectamine RNAiMAX was used to perform siRNA knockdown according to the manufacturer’s instructions (Life Technologies). HUVECs were seeded into 12-well plates 24 h prior to transfection and allowed to grow at 60–80% confluence. Cells were transfected with 15 pmol Trx2 siRNA (Santa cruz, sc-44173) or 3′-UTR Trx2 siRNA (Integrated DNA Technologies, hs.Ri.TXN2.13.1, hs.Ri.TXN2.13.4, and hs.Ri.TXN2.13.5). The transfected cells were grown for 6 h before adding EGM-2 complete culture medium for an additional 48 h prior to harvest.

Several lentiviruses were generated (pLVX-Trx2-WT, pLVX-Trx2-SIM) for overexpression of Trx2 and Trx2-SIM in HUVECs, while pLVX was used as negative control. Lentivirus vectors expressing V5-taged Trx2 and Trx2-SIM were generated by insertion of the corresponding cDNAs into the multicloning sites of the lentivirus backbone vector pLVX-IRES-Neo. The constructs were co-transfected with packaging plasmid (pCMV-R8.2) and envelope plasmid (VSV-G) in to 293T cells for lentivirus package and amplification. HUVECs were infected for 12 h with different lentivirus with polybrene (8 μg/ml). The infections were followed by adding EGM-2 complete culture medium for an additional 48 h before performing assay.

### Protein Extraction, Western Blotting Analysis

Cells were washed with PBS and lysed with RIPA buffer with protease inhibitors (Protease Inhibitor Cocktail, Sigma-Aldrich). Protein extracts were resolved in polyacrylamide gels and analyzed by immunoblotting for the indicated proteins.

### Senescence-Associated β-Galactosidase (SA-β-Gal) Staining

Levels of senescence in cultured HUVECs were evaluated by quantifying the activity of SA-β-gal using a senescence β-galactosidase Staining kit (Cell Signaling Technology, #9860) following the manufacturer’s instruction. HUVECs infected with several different lentiviruses (pLVX, pLVX-Trx2-WT, pLVX-Trx2-SIM), respectively, were treated with or without H_2_O_2_ (150 μM, 6 h) before staining. Cells were washed one time in PBS, fixed for 15 min with fixative solution buffer at room temperature, and washed two times with PBS. Then cells were incubated overnight with freshly prepared SA-β-gal staining solution at 37°C in a dry incubator without CO_2_. Cells were counted in five randomly fields, and the percentage of cells positive for SA-β-Gal was quantified to represent the SA-β-gal activity in three independent experiments.

### Site-Directed Mutagenesis

PCR-based site-directed mutagenesis was carried out using DNA Polymerase KOD-Plus- Neo (TOYOBO CO., LTD., KOD-401), and then the products were digested by *Dpn*I (NEB, R0176S). DH5α competent cells were transformed with the resultant plasmids. The plasmids were amplified, and the mutations were confirmed by sequencing.

### Mitochondrial ROS Measurement

Mitochondrial ROS were measured using MitoSOX^TM^ Red mitochondrial superoxide indicator (Invitrogen, M36008) following the manufacturer’s instructions. Briefly, Hela cells were plated in a glass bottom 6-well plate (MatTek Corporation), treated with 150 μM H_2_O_2_ for 8 h and then stained with 20 nM Mito Tracker Green FM (Invitrogen, M7514) and 10 μg/ml Hoechst and 5 μM MitoSOX for 10 min in complete medium. After removing the reagents and washing three times with warm PBS, fluorescence was detected with a fluorescent microscope (Zeiss Microscope Axiovert 200M).

### Indirect Immunofluorescence Staining

Cells were transfected with plasmids as indicated in individual experiments. Cells were washed with PBS for two times, fixed in 4% PFA for 15–20 min. The fixation solution was discarded and the cells were rinsed with PBS 3 times. Cells were permeabilized in 0.1% TritonX-100 in PBS for 1 min, and then washed with PBS 3 times and blocked by 1% BSA. Cells were incubated with primary antibodies (Trx2 and TFAM) overnight at 4°C, then washed with PBS three times and incubated with secondary antibodies at room temperature for 1 h. After secondary antibody incubation, cells were extensively washed three times in PBS and mounted in Mounting medium with DAPI.

### Immunogold Electron Microscopy

Immunogold electron microscopy was performed as described previously (Zhang et al., 2004). V5 antibody (Cell Signaling Technology, 13202) was used in this experiment.

### Co-immunoprecipitation Assay

Human Umbilical Vein Endothelial Cells were used for endogenous PMPCA SUMOylation coimmunoprecipitation assays. Briefly, cells were washed with PBS and lysed in SUMOylation lysis buffer (Qiu et al., 2017) (50 mM Tris–HCl, pH7.5, 150 mM NaCl, 1% TritonX-100, 1 mM EGTA, 1 mM EDTA, 1× EDTA-free protease inhibitor cocktail and 20 mM NEM). Cell lysates were centrifuged (12 000 g for 20 min at 4°C), and supernatant was collected. Then the supernatant was immediately incubated with 10 μl protein A/G-agarose beads at 4°C for 1 h to preclear. Then the supernatant was incubated with Anti-PMPCA antibody (Santa Cruz, sc-390471) at 4°C for 3 h. Then 20 μl protein A/G beads was added to the antibody-lysate mix and incubated at 4°C for 3 h. Beads were washed with 1 ml of lysis buffer for 5–7 times, then dissolved in 40 μl of SDS-PAGE loading buffer and analyzed by immunoblotting.

### Statistics

Statistical analysis was performed using Prism software program (GraphPad Software, San Diego, CA, United States). Quantitative data are presented as average ±SE, with an indication of the number of samples for each experiment in the text or the corresponding. Unless specified in the text or legends, comparisons between two groups were made using unpaired *t*-test. Statistical significance is shown as ^∗^*p* < 0.05; ^∗∗^*p* < 0.01; ^∗∗∗^*p* < 0.001; ^****^p < 0.0001.

## Results

### Senescence Stimuli Impair Trx2 Processing

**FIGURE 1:**
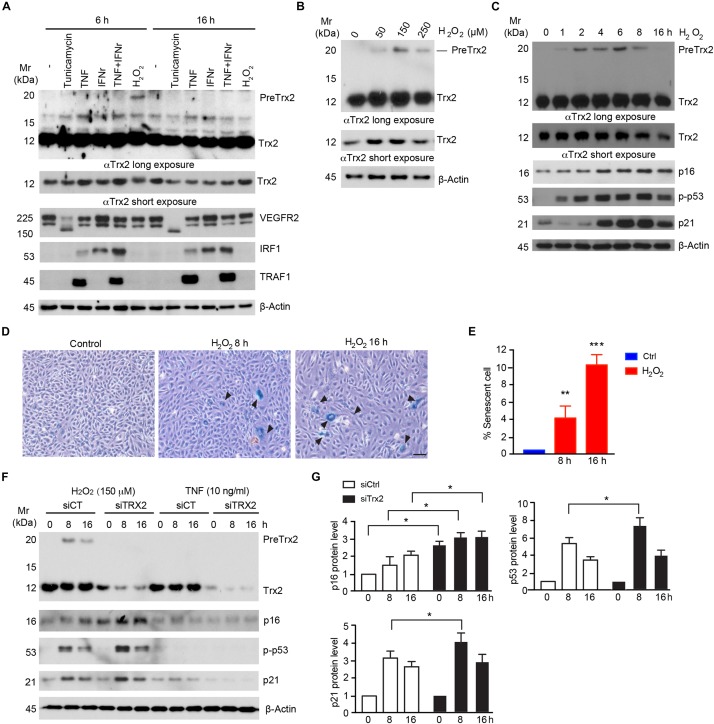
Senescence stimuli impair Trx2 processing. **(A)** Human Umbilical Vein Endothelial Cells HUVECs were incubated with various stress stimuli as indicated. Total cell lysates were subjected to Western blotting with antibodies for specific stress response markers (VEGFR2, IRF1, and TRAF1) as well as Trx2 protein. Trx2 precursor (PreTrx2) and mature Trx2 (Tx2) are indicated. A shorter exposure and a longer exposure for Trx2 are shown. **(B)** Effects of H_2_O_2_ on Trx2 processing. HUVECs were incubated with different doses of H_2_O_2_ as indicated. Trx2 protein was determined by Western blotting. **(C–E)** Kinetics of H_2_O_2_ on Trx2 processing and senescence. HUVECs were incubated with H_2_O_2_ at 150 μM for indicated time. Trx2 protein and senescence markers (p16, p-p53, and p21) were determined by Western blotting with specific antibodies **(C)**. Senescence-associated β-galactosidase assays for H_2_O_2_-treated cells (8 h and 16 h). Representative images are shown in **(D)** with quantifications in **(E)**. Arrowheads pointed to the positive cells. Data are mean ± SEM from three independent experiments. ^∗^*P* < 0.05; ^∗∗^*P* < 0.01; and ^∗∗∗^*P* < 0.001. **(F,G)** Trx2 protects cellular senescence. HUVECs transfected with control siRNA or Trx2 siRNA followed by treatment with H_2_O_2_ or TNF. Trx2 protein and senescence markers (p16, p-p53, and p21) were determined by Western blotting **(F)**. Relative protein levels were quantified by taking untreated siCtrl as 1.0 **(G)**. Data shown are representative of three experiments. Scale bar: 100 μm **(D)**. Mr: molecular weight.

Trx2 has a mitochondrion targeting sequence (MTS) at its N-terminus (Damdimopoulos et al., 2002), and the predicted molecular weight of Trx2 precursor (PreTrx2) and mature Trx2 are 19 kDa and 12 kDa, respectively. To explore if Trx2 processing is altered under pathological conditions, we examined Trx2 processing in response to various stress stimuli associated with vascular diseases. To this end, HUVECs were treated with proinflammatory cytokines (TNF, IFN-γ or combo), ER stress activator (tunicamycin), and senescence stimuli (H_2_O_2_ or menadione) (Debattisti et al., 2017). Among these factors, only senescence stimuli, including H_2_O_2_ (Figure 1A) and menadione (Supplementary Figure 1), induced an accumulation of unprocessed Trx2 which was 19 kDa (PreTrx2) above the mature form (12 kDa). Known effects of TNF (induction of TRAF1), IFN-γ (induction of IRF1) and tunicamycin (deglycosylation of VEGFR2) were evident by Western blotting. However, these factors, unlike H_2_O_2_ or menadione, did not alter the Trx2 processing (Figure 1A).

Senescence-associated β-galactosidase, along with cell cycle inhibitor p16, are regarded to be biomarkers of cellular senescence. Activation of p53 and upregulation of its downstream target p21 have also been associated with cellular senescence (Sharpless and Sherr, 2015). We further examined effects of different doses and time courses of H_2_O_2_ on Trx2 processing. H_2_O_2_ at 150 μM induced a maximum accumulation of preTrx2 at 2–6 h (Figures 1B,C). Interestingly, the kinetics of PreTrx2 accumulation correlated with activation of senescence markers, including expression of SA-β-Gal, p16, phosphorylation of p53 which preceded the induction of p21 (Figures 1C–E). The observation that senescence stimuli alter Trx2 processing suggests that Trx2 may normally protect cellular senescence. To test this possibility, Trx2 was knocked down by siRNAs in HUVECs followed by treatment with H_2_O_2_-induced for 6–16 h. TNF was used as non-senescence inducer control. Trx2 knockdown alone did not induce senescence in ECs, but augmented H_2_O_2_-induced activation of EC senescence as evident by increased expression of p16 and p21, and phosphorylation of p53 (Figure 1F). These results indicate that Trx2 protects EC from senescence, and senescence stimuli impair Trx2 processing during cellular senescence.

### Mapping the Residues Critical for Trx2 Processing

**FIGURE 2:**
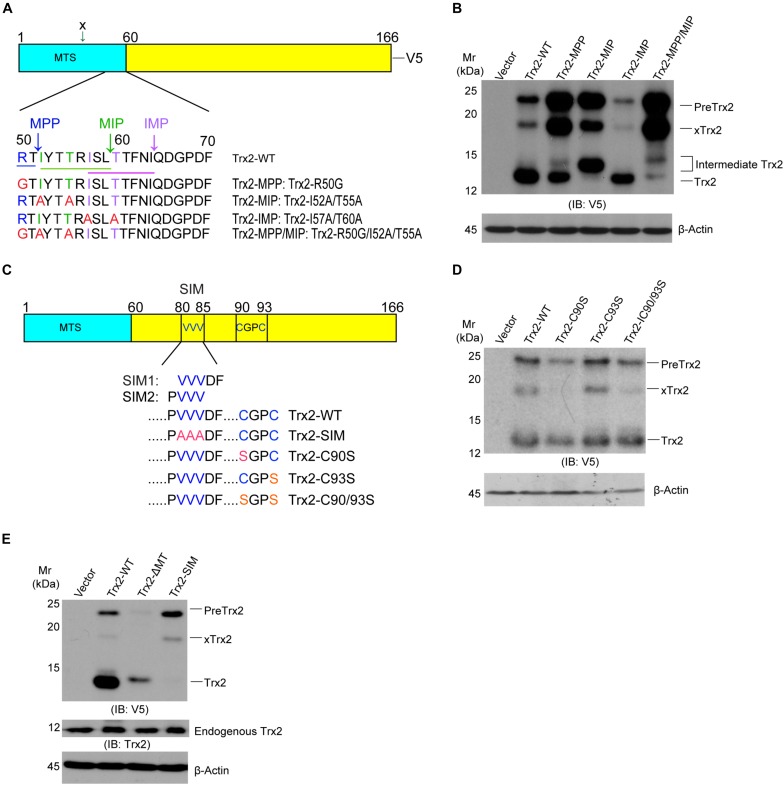
Identification of critical motifs for Trx2 processing. **(A)** Sequence analyses of the mitochondrial targeting sequence (MTS) on Trx2 protein. The predicted conserved motif (underlined) and cleavage sites (vertical arrows) of mitochondrial processing peptidases (MPP), mitochondrial intermediate peptidase (MIP) and inner membrane peptidase (IMP) are shown. The conserved amino acid residues for each motif are indicated by different colors. **(B)** MPP/MIP sites are critical for Trx2 processing. pcDNA3.1 empty vector, Trx2-WT and various mutants were transfected into cells. Cell lysates were subjected to Western blotting with anti-V5. Pre-Trx2, mature Trx2 as well as processing intermediates are indicated. xTrx2: 18 kDa band is resulted from a cleavage within the MTS before the MPP site by an unknown enzyme. **(C)** Sequence analyses of Trx2 mature protein. The SUMO interaction motif (SIM) (VVVDF or PVVV), the catalytic active site (C^90^GPC^93^), and respective mutations are indicated. **(D,E)** The SIM, but not the catalytic site, is critical for Trx2 processing. pcDNA3.1 empty vector, Trx2-WT and various mutants were transfected into cells and the resultant cell lysates were subjected to Western blotting. Various Trx2 protein forms are indicated. Data shown are representative of three experiments.

Mitochondrial protein precursors, depending on their final destination in the mitochondria, are processed in sequential steps by mitochondrial processing peptidase (MPP) and mitochondrial intermediate peptidase (MIP) cleavage site of R-X ↓(F/L/I)-X-X-(T/S/G)-X-X-X-X↓ (Horwich et al., 1985; Gakh et al., 2002). The MTS of Trx2 contains a potential (V/I)-X-X-T-X-X-X-X↓ motif that can be recognized by inner membrane peptidase (IMP) between Ile-64 and Gln-65 (Hirasaka et al., 2011; Figure 2A). To verify the role of these motifs in Trx2 processing, we mutated the conserved amino acid of the three motifs within the Trx2 MTS in an expressing system in which Trx2 contained a V5-tag at the C-terminus. Upon overexpression in HUVECs, Trx2-WT (with V5-tag) was present as two major bands, a mature form at 14 kDa and PreTrx2 at 21 kDa. An extra band at 18 kDa was also detected and likely resulted from a cleavage before the MPP site by an unknown mechanism (the site is marked as x in Figure 2A and the resultant protein is indicated by xTrx2 in Figure 2B). Mutation at the MPP site alone (Trx2-MPP) reduced mature Trx2 with increased Trx2 precursors (PreTrx2 and xTrx2). Similarly, mutations at the MIP site (Trx2-MIP) diminished mature Trx2 with increased Trx2 precursors. Interestingly, an intermediate form above mature Trx2 was detected in Trx2-MIP; this band was likely resulted from a cleavage at the upstream MPP site, generating a form with an extra 13 amino acid longer than the mature Trx2. Mutations at both MPP and MIP motifs (Trx2-MPP/MIP) completely blocked the Trx2 processing, accumulating the Trx2 precursors (PreTrx2 and xTrx2). On the contrary, mutation at the IMP site (Trx2-IMP) did not increase Trx2 precursors (Figure 2B). These results suggested that both MPP and MIP cleavage sites, but not the IMP site, were critical for the Trx2 processing in the mitochondria.

Some mitochondrial proteins contain a sorting signal outside the MTS (Monaghan and Whitmarsh, 2015). This promoted us to examine if other sequens are involved in Trx2 processing. Trx2 contains a catalytic active site Trx2 contains the redox-active site (C^90^GPC^93^). A reduced active form of Trx2 in mitochondria undergoes reversible oxidation to the Cys disulfide (Trx-S2) upon reducing protein substrate and is then regenerated by Trx reductase-2 (TrxR2) at the expense of NAPDH (Holmgren, 2000). Moreover, it is now recognized that Cys residues could be undergo various modifications, include sulfenylation and disulfide formation, formation of higher oxidation states, S-nitrosylation, and other modifications (Go et al., 2015). These modifications may potentially affect Trx2 folding and processing. Besides the redox-active site, Trx2 contains a SUMO interaction motif (SIM) (SIM1: 81-85 VVVDF) or (SIM2: 80-83 PVVV) based on SIM predictions using GSP-SUMO (Zhao et al., 2014) and JASSA (Beauclair et al., 2015; Figure 2C). To determine if these motifs are involved in Trx2 processing, we mutated these sites to generate Trx2-C90S, C93S, C90/93S and Trx2-SIM mutants. Mutations at the catalytic site C90/C93 had no effect on Trx2 processing (Figure 2D). To our surprise, Trx2-SIM existed only as the Trx2 precursor form. Compared to WT and Trx2-ΔMT, which lacks the MTS and expresses as a mature form, Trx2-SIM completely lacks the mature protein (Figure 2E). These results suggest that the SIM motif is critical for the Trx2 processing.

### SUMO Interaction Motif (SIM) Mutation Has No Effect on Trx2 Localization

**FIGURE 3:**
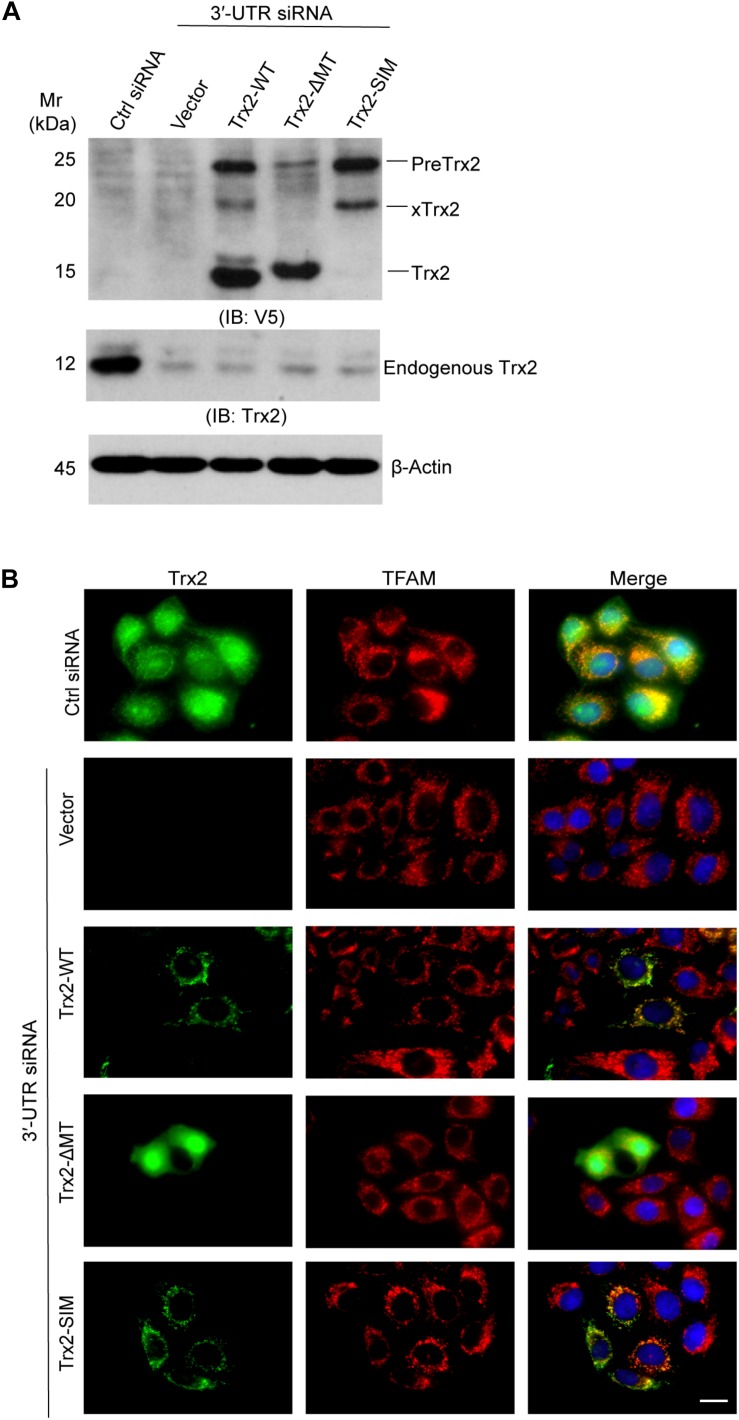
SUMO interaction motif (SIM) has no effect on Trx2 localization. Endogenous Trx2 in EC was knocked down by Trx2 3′-UTR siRNA, and wild type Trx2, Trx2-ΔMT or Trx2-SIM was re-expressed in cells. **(A)** Cell lysates were subjected to Western blotting with anti-V5 and anti-Trx2. Endogenous Trx2 and various forms of overexpressed Trx2 proteins are indicated. **(B)** Trx2 localization was determined by indirect Immunofluorescence staining with anti-Trx2 and anti-TFAM. Scale bar: 25 μm **(B)**. Data shown are representative of three experiments.

Since the processing of mitochondrial precursor proteins are associated with their localization, we first examined if the SIM influences Trx2 localization. To exclude the influence of endogenous Trx2, we first knocked down endogenous Trx2 using a Trx2 3′-UTR siRNA in ECs followed by re-expression of wild type Trx2 or Trx2-SIM. Trx2-ΔMT lacking the MTS was used as a cytosolic protein control. Western blotting showed these mutants were reconstituted into EC efficiently (Figure 3A). Trx2 localization was determined by indirect Immunofluorescence staining. Consistent with the Western blotting results, endogenous Trx2 was completely removed by the Trx2 3′UTR siRNA. As expected, re-expressed Trx2-WT was localized inside mitochondria where it was colocalized with the mitochondrial transcription factor TFAM, whereas Trx2-ΔMT is homogeneous distributed in the cytoplasm as previously reported (Damdimopoulos et al., 2002). To our surprise, Trx2-SIM, similar to Trx2-WT, completely colocalized in the mitochondria (Figure 3B). These results reveal that Trx2 SIM has no effect on Trx2 localization but blocks subsequent Trx2 processing inside the mitochondria.

### The Role of MPP SUMOylation in Trx2 Processing

**FIGURE 4:**
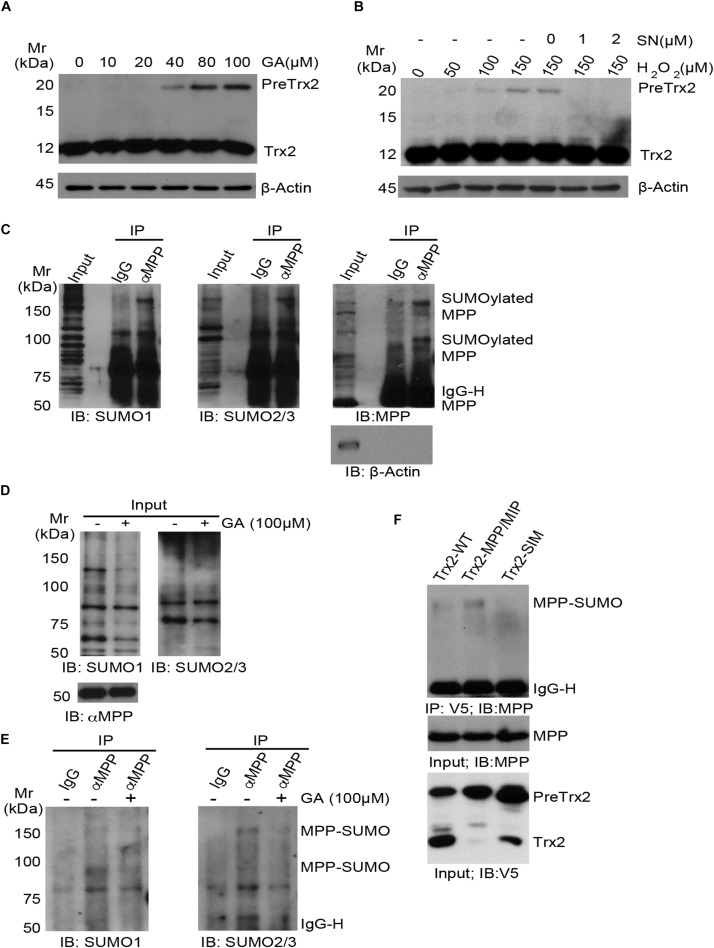
The role of MPP protein SUMOylation in Trx2 processing. **(A,B)** Effects of SUMOylation on Trx2 processing. HUVECs were treated with different concentrations of Ginkgolic Acid (GA) at 0–100 μM for 4 h **(A)**, or treated with H_2_O_2_ for 6 h with or without Streptonigrin (1 or 2 μM) **(B)**. Trx2 protein was determined by immunoblotting. Trx2 precursor (Pre-Trx2) and mature Trx2 (Tx2) are indicated. **(C)** SUMOylation of endogenous α-MPP in HUVECs. HUVEC lysates were subjected to co-immunoprecipitation (co-IP) with anti-αMPP followed by immunoblotting with anti-SUMO1, anti-SUMO2/3 and anti-αMPP, respectively. MPP and SUMOylated MPP are indicated. **(D,E)** GA blocks MPP SUMOylation. HUVEC were untreated or treated with GA (100 μM) for 4 h. Total SUMO1, SUMO2/3 and αMPP were determined by immunoblotting **(D)**. SUMOylation of MPP was detected as in **C (E)**. **(F)** HUVECs were infected with V5-tagged Trx2-WT, Trx2-MPP/MIP and Trx2-SIM. Association of Trx2 with endogenous MPP was determined by a co-immunoprecipitation assay with anti-V5 (Trx2) followed by immunoblotting with anti-MPP. SUMOylated MPP are indicated. All experiments were repeated three times.

Given to the potential role of SIM in Trx2 processing, we examined the role of protein SUMOylations in Trx2 processing. Ginkgolic Acid [GA, 2-hydroxy-6-(8-pentadecenyl)] could specifically and directly bind to SUMO-activating enzyme E1, blocking formation of the E1-SUMO1 intermediate (Fukuda et al., 2009). On the contrary, streptonigrin (SN), a natural product isolated from *Streptomyces flocculus*, acts as an inhibitor of SENPs by disrupting SENP1-SUMO1 interaction, with higher inhibitory effect against SENP1 than other SENPs (Ambaye et al., 2018). Its inhibitory effect on SUMO-2/3 was also detected in different cell lines (Liu et al., 2018; Qiu et al., 2018). We first examined effects of GA on Trx2 processing. HUVECs were treated with different concentrations of GA (0–100 μM) for 4 h. We found that GA greatly induced an accumulation of PreTrx2 in a dose-dependent manner (Figure 4A). We then used SN as a SUMOylation agonist to see if SN could reverse H_2_O_2_ induced Trx2 unprocessing. HUVECs were treated with H_2_O_2_ in the presence or absent of SN. Results showed that SN completely diminished H_2_O_2_-induced PreTrx2 accumulation (Figure 4B). These data indicated that chemical inhibition of protein SUMOylation attenuated, while inhibition of SENP1-mediated deSUMOylation (increases of protein SUMOylation) promoted, the Trx2 processing.

We hypothesized that Trx2 processing enzymes (MPP and MIP) could be SUMOylated to facilitate the Trx2 processing. MPP is a heterodimeric enzyme responsible for the first cleavage of the mitochondrial targeting sequence. PMPCA (9q34.3) encodes α-MPP, the α-subunit of mitochondrial processing peptidase (MPP; also named as PMPCA) (Gakh et al., 2002). It is reported that α-MPP can be SUMOylated at multiple sites by mass-spectrometry (Hendriks et al., 2017, 2018), therefore, we first examined if endogenous α-MPP could be SUMOylated in HUVECs by co-immunoprecipitation assays with anti-MPP followed by immunoblotting with anti-SUMO1 and SUMO2/3. Results showed that α-MPP (but not β-actin) was detected in the immunoprecipitation, and at least two higher bands (∼80 kDa and ∼160 kDa) were detected in both SUMO1 and SUMO2/3 blots. However, these bands were absent in the IgG co-immunoprecipitation. Based on the size of α-MPP (∼50 kDa), α-MPP is likely SUMOylated at multiple sites by both SUMO1 and SUMO2/3 (Figure 4C). We further tested if GA could suppress SUMOylation of MPP by treating HUVEC with GA (100 μM) for 4 h. GA had no effect on total level of MPP but diminished both MPP-SUMO1 and MPP-SUMO2/3 (Figures 4D,E).

Finally, we tested if Trx2 via SIM directly interacts with SUMOylated MPP. HUVECs were infected with V5-tagged Trx2-WT, Trx2-MPP/MIP and Trx2-SIM. Association of Trx2 with endogenous MPP was determined by a co-immunoprecipitation assay with anti-V5 (Trx2) followed by immunoblotting with anti-MPP. We found that Trx2-WT weakly, while Trx2-MPP/MIP strongly, associated with SUMOylated MPP. In contrast, Trx2-SIM lost the ability to associate with SUMOylated MPP (Figure 4F). The stronger binding of Trx2-MPP/MIP with SUMOylated MPP suggest that Trx2-MPP/MIP acts as a “substrate trapping” mutant (i.e., with enzyme binding without cleavage). Taken together, these results indicated that SUMOylated MPP, by binding to the Trx2 SIM motif, mediated the Trx2 cleavage at the MLS.

### Trx2-SIM Loses Anti-ROS and Anti-senescence Activities

**FIGURE 5:**
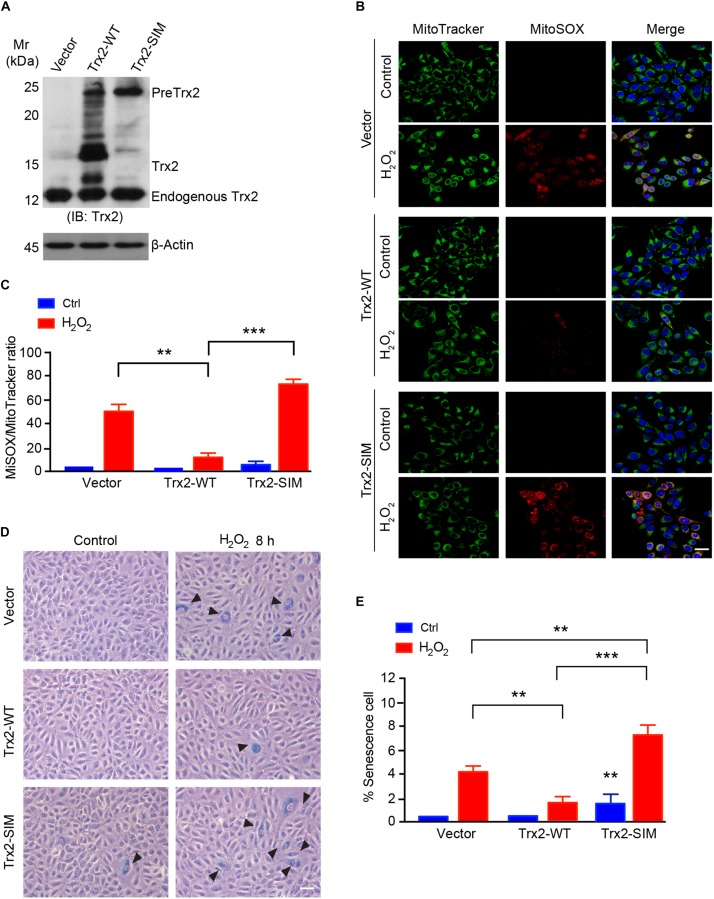
Trx2-SIM loses anti-ROS and anti-senescence activities. **(A)** Trx2 expression. Cells were infected with lentivirus expressing Trx2 or Trx2-SIM. Trx2 expression was verified by Western blotting with anti-Trx2. PreTrx2, Trx2 from overexpressed Trx2-WT and Trx2-SIM as well as endogenous Trx2 are indicated. **(B,C)** Effects on ROS generation. Trx2-WT or Trx2-SIM-expressing cells were treated with H_2_O_2_ at 150 μM for 2 h followed by staining with 20 nM MitoTracker Green, 5 μM MitoSOX Red and 10 μg/ml Hoechst for 30 min. Fluorescence images were captured and ratio of MitoSOX Red-positive vs MitoTracker Green-positive cells were quantified. **(D,E)** Effects on senescence cells. Representative images of SA-β-Gal-stained cells **(D)** with quantification of % senescence cells **(E)**. Arrowheads pointed to the positive cells. Data are mean ± SEM from three independent experiments. ^∗∗^*P* < 0.01; ^∗∗∗^*P* < 0.001. Scale bar: 25 μm **(B)**, 100 μm **(D)**.

Our previous and present data suggest that Trx2 is responsible for suppressing ROS (Dai et al., 2009; Huang et al., 2015) and ROS-induced senescence (see Figure 1). We examined if Trx2-SIM, localizing in mitochondria without processing, retained the anti-ROS and anti-senescence activities of Trx2. To this end, HUEVCs were overexpressed for Trx2-WT or Trx2-SIM by a lentivirus expressing system. Expression of Trx2 was verified by Western blotting (Figure 5A). Cells were untreated or treated with mitochondrial ROS-inducer H_2_O_2_ which strongly induced elevated mitochondrial ROS as measured by a MitoSOX Red fluorescence probe. Overexpression of Trx2-WT attenuated H_2_O_2_ induced mitochondrial ROS. In contrast, overexpression of Trx2-SIM slightly increased basal mitochondrial ROS and significantly augmented H_2_O_2_-induced ROS generation (Figure 5B with quantification in Figure 5C).

To further determine if Trx2-SIM affects the anti-senescence function of Trx2, we analyzed the senescence response to H_2_O_2_ inTrx2-WT or Trx2-SIM-expressing cells by SA-β-Gal assay. Consistent with the anti-ROS activity, Trx2-WT suppressed H_2_O_2_ induced senescence. Expression of Trx2-SIM not only weakly activated basal SA-β-Gal activity, but also strongly augmented H_2_O_2_ induced cellular senescence (Figures 5D,E). Taken together, these data suggest that Trx2-SIM loses the ROS-scavenging and anti-senescence activities.

## Discussion

In the present study, we demonstrate that Trx2 protects endothelial cells from senescence, and senescence stimuli such as H_2_O_2_ impair Trx2 processing during cellular senescence. Mechanistically, Trx2 processing is mediated by MPP and MIP-recognition sites within the MTS. Interestingly, SUMO-interacting motif (SIM) within the mature Trx2 protein is also necessary for Trx2 processing with no effect on Trx2 mitochondrial targeting. Notably, inhibition of protein SUMOylation confers an elevation of unprocessed Trx2, suggesting protein SUMOylation facilitates the Trx2 processing. Moreover, the unprocessed form of Trx2 is unable to protect cells from both ROS generation and oxidative stress-induced senescence. Our study identifies a novel mechanism of ROS regulating Trx2 in cellular senescence via SUMOylation.

As one of the most studied mitochondrial protein, Trx2 is a small redox protein, ubiquitously presented in tissues with high metabolic activity, such as liver, brain and heart.Trx2 not only protects against oxidative stress in mitochondria, but also induces the cells insensitive to ROS-induced apoptosis by regulating apoptosis related molecules and critical transcription factors, such as apoptosis signal-regulating kinase (ASK1) and nuclear factor kappa B (NF-_k_B) (Zhang et al., 2004; Dai et al., 2009). Trx2 is able to inhibit ASK1-induced apoptosis in both endothelium cells and cardiomyocytes. Moreover, reduced Trx2 expression, elevated levels of phosphorylated ASK1 and activated caspase-3 is found in cardiomyocytes of patients with dilated cardiomyopathy (DCM) compared to that of healthy organ donors (Huang et al., 2015). However, the role of Trx2 in senescence has not been explored.

In the present study, for the first time, we revealed a novel anti-senescence function of Trx2 in endothelial cells. While oxidative stress triggered by H_2_O_2_ can lead to premature senescence (Toussaint et al., 2000; Huo et al., 2018), Overexpression of Trx2 prevents, whereas knockdown of Trx2 augments H_2_O_2_-induced endothelial senescence, indicated by SA-β-gal activity and the expression of senescence-related markers, including p-p53, p21, and p16. In supporting our finding, it has been reported that Trx2-intereacting protein (TXNIP) can translocate to the mitochondria where it binds to and oxidizes Trx2, leading to mitochondrial dysfunction (Saxena et al., 2010). Furthermore, TXNIP promotes NLRP3 inflammasome activation in senescent endothelial cells (Yin et al., 2017),possibly via inhibiting Trx2 activity. The molecular mechanism by which Trx2 inhibits endothelial senescence remains to be further elucidated.

We have identified several unique motifs within Trx2 are critical for its mitochondrial processing and antioxidant activity. The positively charged MTS is known to be essential for its mitochondrial anchoring (Gakh et al., 2002; Carapito et al., 2017), which is followed by the motifs cleaved by MPP, MIP, and/or IMP for mitochondrial protein processing. We show that both MPP and MIP motifs mediate the Trx2 processing. Mutations at the MPP and MIP sites, but not the IMP site, diminish the mature Trx2 along with increased Trx2 precursors. These results are consistent with the notion that IMP is usually involved in maturation of mitochondrial proteins delivered to the intermembrane space, while MPP and MIP are responsible maturation of matrix proteins such as Trx2.

**FIGURE 6:**
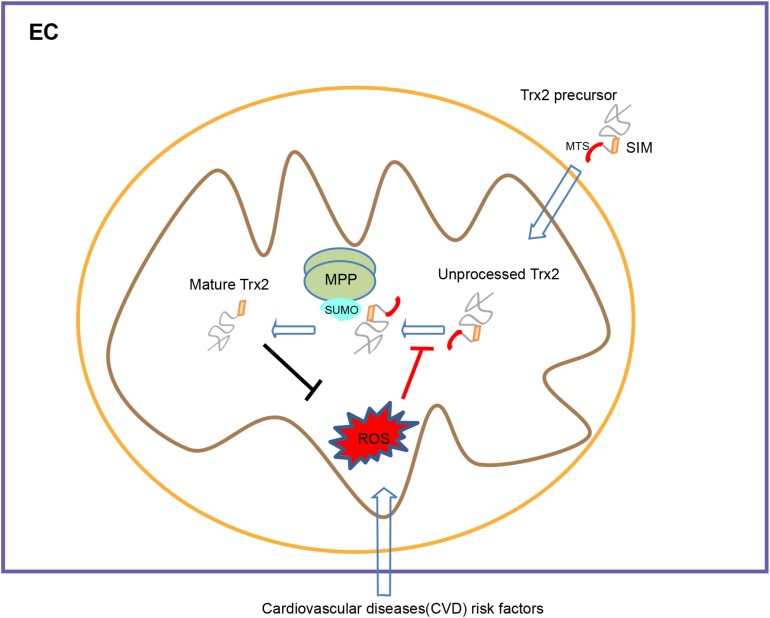
A model for the role of SIM in Trx2 processing and function. Trx2 processing is mediated by mitochondrial processing peptidase (MPP) and mitochondrial intermediate peptidase (MIP)-cleavage sites within the MTS. A SUMO interaction motif (SIM) within the mature Trx2 protein, although not involved in mitochondrial targeting, is critical for Trx2 processing. The α–MPP subunit is a SUMOylated protein that potentially recruits Trx2 to MPP complex via binding to the SIM of Trx2 for subsequent cleavage. Cardiovascular diseases (CVD) risk factors lead to ROS generation and blocks Trx2 processing, leading to endothelial senescence and CVD. SIM, SUMO interaction Motif; MTS, mitochondria targeting sequence; MPP, Mitochondrial processing peptidase.

The most significant finding in our study is that we have defined the mechanism by which protein SUMOylation regulates the Trx2 processing. First, SUMOylation inhibitor confers an elevation of unprocessed Trx2, whereas the SUMOylation agonist reverses H2O2-induced Trx2 unprocessing, which correlates with their effects on the cellular senescence. Secondly, we unexpectedly identified that a SIM motif within the mature Trx2 is essential for Trx2 processing and antioxidant activity. Trx2 contains a potential SIM based on sequence analyses, and mutation at the SIM motif completely blocks Trx2 processing without disturbing Trx2 mitochondrial targeting. Interestingly, Trx2-SIM blocks the cleavages at both MPP and MIP sites, suggesting that the Trx2 SIM domain (via binding to SUMOs) is critical for Trx2 processing in the mitochondria. This promoted us to examine if MPP or/and MIP themselves are SUMOylated. Thirdly, our data indicate that endogenous α-MPP, the enzyme that cleaves the presequence of Trx2, is SUMOylated by both SUMO1 and SUMO2/3 at multiple sites. More importantly, either Trx2-WT or a substrate trapping mutant Trx2-MPP/MIP, but not Trx2-SIM, associate with SUMOylated MPP. We propose that α-MPP binds the SIM motif of Trx2 and recruits Trx2 to the MPP complex for its subsequent cleavage (Figure 6: A model for the role of SIM in Trx2 processing and function). It needs to further examine if MIP is also SUMOylated and if the SUMOylation modulates enzymatic activities of MPP and MIP during Trx2 processing.

Our study provides a novel, unexpected link between cellular senescence and mitochondrial protein processing. We find that senescence stimuli induce endothelial cell senescence concomitant with an attenuation of mitochondrial Trx2 processing, resulting in accumulation of Trx2 precursor. Of note, one report suggest that Trx2 mitochondrial processing could be regulated by uncoupling protein 3 (UCP3) via interaction with the MTS of Trx2 (Hirasaka et al., 2011). Although Trx2 processing is complete, the resultant processed Trx2 is not imported to mitochondrial matrix, is rather localized in the intermembrane space where Trx2 may mitigate oxidative stress induced during pathological conditions (Hirasaka et al., 2011). Further studies are necessary to investigate the regulation and function of Trx2 processing during cellular senescence in aging and cardiovascular diseases.

## Data Availability

The raw data supporting the conclusions of this manuscript will be made available by the authors, without undue reservation, to any qualified researcher.

## Author Contributions

All authors designed and performed the study and analyzed the data. CC, YC, and WM wrote the manuscript.

## Conflict of Interest Statement

The authors declare that the research was conducted in the absence of any commercial or financial relationships that could be construed as a potential conflict of interest.
