# Supplementary material for: A Unique SUMO-Interacting Motif of Trx2 Is Critical for Its Mitochondrial Presequence Processing and Anti-oxidant Activity
Source: Front Physiol. 2019 Aug 27;10:1089. doi: 10.3389/fphys.2019.01089 (PMC6727865; doi:10.3389/fphys.2019.01089)
Supplement: Supplementary file 1 [file Data_Sheet_2.PDF]

**Supplemental Figure**

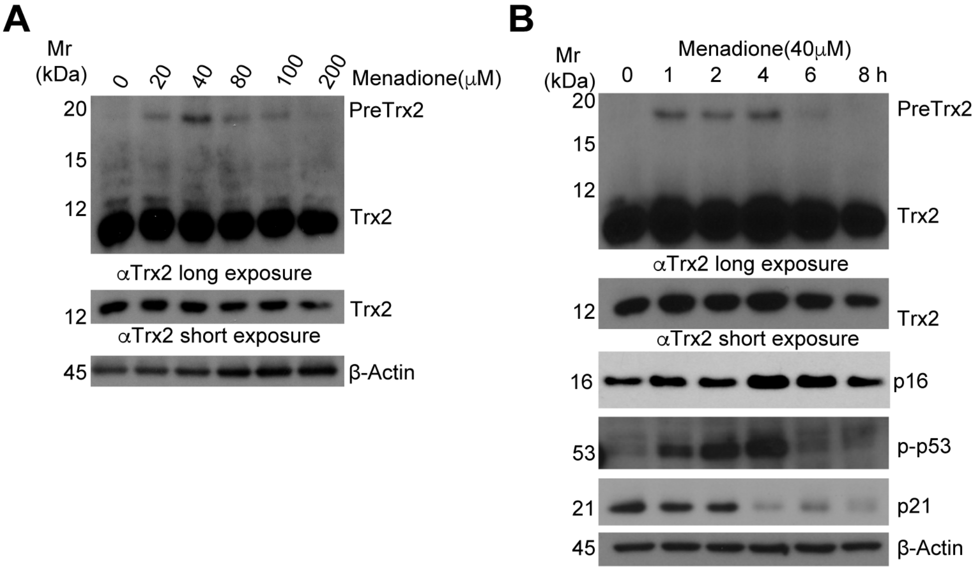


**Supplemental Figure 1: Senescence stimuli impair Trx2 processing. (A)** Effects of different doses of menadione on Trx2 processing. HUVECs were incubated with different doses of menadione as indicated. Trx2 protein was determined. **B.** Kinetics of menadione on Trx2 processing and senescence. HUVECs were incubated with menadione at 40 μM for indicated time. Trx2 protein and senescence markers (p16, p-p53, and p21) were determined by Western blotting with specific antibodies (C).
